# Dynamic Alteration of the Colonic Microbiota in Intestinal Ischemia-Reperfusion Injury

**DOI:** 10.1371/journal.pone.0042027

**Published:** 2012-07-27

**Authors:** Fan Wang, Qiurong Li, Chenyang Wang, Chun Tang, Jieshou Li

**Affiliations:** Research Institute of General Surgery, Jinling Hospital, School of Medicine, Nanjing University, Nanjing, China; Charité, Campus Benjamin Franklin, Germany

## Abstract

**Background:**

Intestinal ischemia-reperfusion (I/R) plays an important role in critical illnesses. Gut flora participate in the pathogenesis of the injury. This study is aimed at unraveling colonic microbiota alteration pattern and identifying specific bacterial species that differ significantly as well as observing colonic epithelium change in the same injury model during the reperfusion time course.

**Methodology/Principal Findings:**

Denaturing gradient gel electrophoresis (DGGE) was used to monitor the colonic microbiota of control rats and experimental rats that underwent 0.5 hour ischemia and 1, 3, 6, 12, 24, and 72 hours following reperfusion respectively. The microbiota similarity, bacterial diversity and species that characterized the dysbiosis were estimated based on the DGGE profiles using a combination of statistical approaches. The interested bacterial species in the gel were cut and sequenced and were subsequently quantified and confirmed with real-time PCR. Meanwhile, the epithelial barrier was checked by microscopy and D-lactate analysis. Colonic flora changed early and differed significantly at 6 hours after reperfusion and then started to recover. The shifts were characterized by the increase of *Escherichia coli* and *Prevotella oralis*, and *Lactobacilli* proliferation together with epithelia healing.

**Conclusion/Significance:**

This study shows for the first time that intestinal ischemia-reperfusion results in colonic flora dysbiosis that follows epithelia damage, and identifies the bacterial species that contribute most.

## Introduction

Intestinal ischemia-reperfusion (I/R) is a frequent phenomenon during intestinal stricture, gangrene, fulminant universal colitis, and mesenteric low flow secondary to other critical diseases, such as trauma, shock and major surgeries, carrying high mortality rate [1], [2]. Diagnosis and management are clinically challenging. The main therapy remains supportive, other than in cases that surgeries are mandatory. Therefore, it is important to investigate the pathophysiological mechanisms that initiate and propagate the intestinal I/R.

The gut luminal flora had been described as “a forgotten organ” [3]. The large bowel is the most heavily colonized part of the gastrointestinal tract, which accommodates nearly 400 different species of bacteria, comprising 10^13^ to 10^14^ microorganisms whose genome is over 100 times as large as human beings’ genome [4], [5]. Majority of gut microbes have a profound influence on human physiology, such as energy and vitamins harvest from food [6], [7], epithelial cell growth and differentiation [8], immune system development and homoeostasis [9], and prevention of noncommensal organisms from expanding [10]. Meanwhile, changes of colonal microbiome are associated with ulcerative colitis [11], infectious colitis [12], colon cancer [13], critical illnesses [14] and small bowel transplantation [15].

One of the essential functions of the intestine is to maintain a barrier which prevents the entry of potentially harmful microorganisms to adjacent and distant sterile organs. The mechanical barrier gets impaired through splanchnic hypoxic and subsequent reperfusion, and results in bacterial translocation [16], [17], [18]. Moreover, most translocating bacteria originate from colonic resident identical ones [19], and the incidence of bacteria translocation is associated with colon [20].

Intestinal microbial community changes have been investigated in critical ill patients and animal models using conventional bacteriological analysis techniques [21], [22], [23], while few study applies culture-independent methods [24]. Since the majority of our indigenous microbial community members are unculturable, it is meaningful to investigate the changes of colonic microbiota in intestinal ischemia and reperfusion injury using molecular techniques. Until now, it has never been elucidated how gut ecology changes according to the reperfusion time course. Furthermore, there is no assessment of both colonic mechanical and bacterial barriers at the same time.

Therefore, we undertook the study (1) to characterize the changes of colonic microbiota in relation to intestinal ischemia-reperfusion using denaturing gradient gel electrophoresis (DGGE) method and (2) to observing both epithelial barrier and gut flora impairment and recovery in the same model longitudinally.

## Results

### Intestinal Ischemia-reperfusion Induces Dynamic Changes of the Colonic Microbiota

#### Temporal changes of colonic community pattern

**Figure 1:**
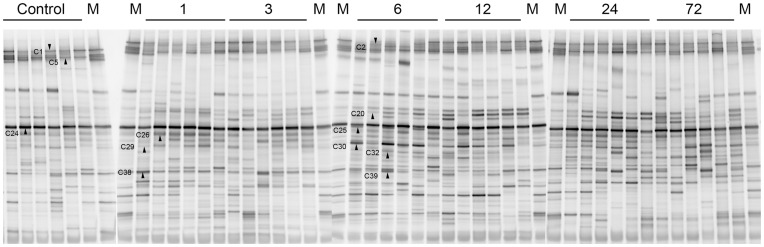
Intestinal ischemia-reperfusion changes the colonic microbiota dynamically shown in DGGE profiles. Sample numbers above lanes indicate hours after reperfusion and M denotes marker lane, which was used for gel to gel comparison. Each band represents a bacterial clone. Band numbers and arrowheads indicate the position of bands excised for sequence analyses. (e.g. “C20” means band C20).

**Figure 2:**
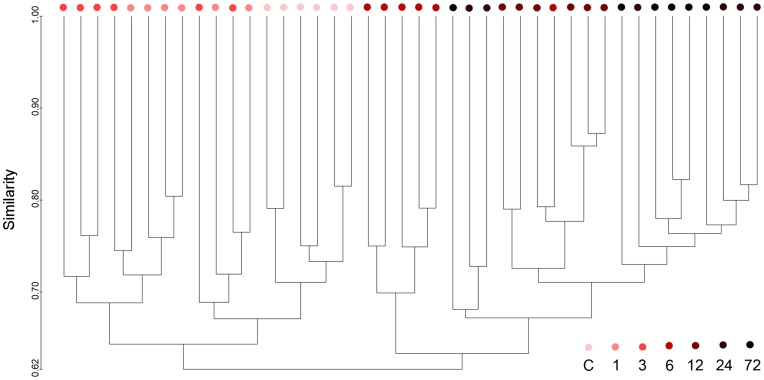
Cluster of colonic microbiota based on DGGE profiles using UPGMA. Metric scale denotes degree of similarity. The numbers and color coded symbols denote samples in a group at the particular hours after reperfusion, while the letter C denotes samples in the control group.

The DGGE profiles of proximal colonic flora from different reperfusion time groups showed shifts of colonic microbial community composition during the reperfusion time (Figure 1). It could be observed that some bands became more intense and appeared more prevalently, such as band C30. However, the changes of the DGGE band profiles were difficult to be quantified by observation. Therefore, we utilized Dice coefficient and UPGMA as a cluster method to demonstrate band pattern similarity (Figure 2). There were two primary clusters. The left cluster contained all normal samples and samples of 1 and 3 hours after reperfusion, whereas the right one contained all other groups of later reperfusion time points. There were variations in DGGE profiles between early injury rats and normal rats. Total similarity of the first cluster was 64%, while similarity of colonic microbiota within the group of normal rats was 71%. Sub-clusters of the right cluster also showed that the composition of the microbiota had a time dependent behavior. Colonic microbiota patterns of 6 hours of reperfusion were distinct from patterns of other later time points, except for only one individual.

**Figure 3:**
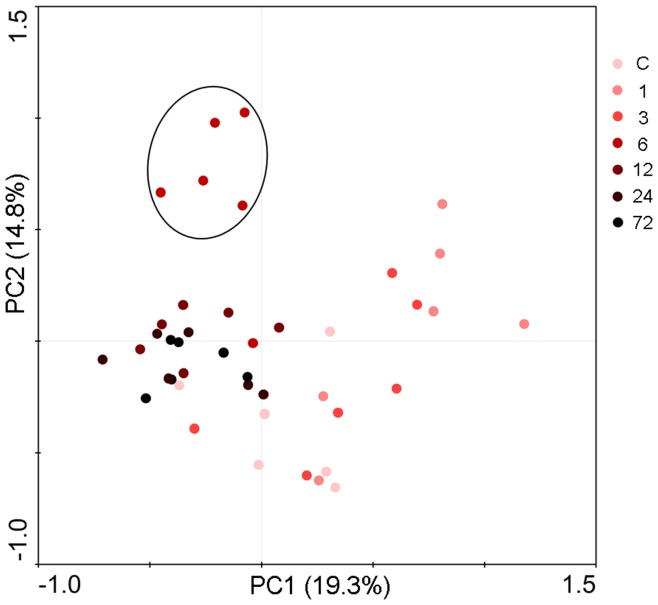
Principal component analysis (PCA) of colonic microbiota based on DGGE fingerprinting. Each color coded symbol represents a single sample according to reperfusion time. Samples grouped in a solid circle represent colonic microbiota of five 6-hr intestinal ischemia-reperfusion rats.

Principal component analysis (PCA) of DGGE fingerprints of colonic microbiota (Figure 3) confirmed and exhibited the temporal changes of colonic flora according to different reperfusion time points more thoroughly. The distance between 2 data points represents the extent of difference between of the 2 rats’ gut microbial compositions. Microbial structure of 1 and 3 hours of reperfusion showed a separation from normal colonal microbiota by PCA axis 1 and 2. Furthermore, 6 hours reperfusion colonic microbiota differed considerably from normal samples by moving towards left of PCA axis 1 and up of PCA axis 2, and described the largest variation (circled in a solid circle upper left in Figure 3). From then on, the composition of gut flora composition began moving back towards normal groups according to the lasting of reperfusion time along axis 2.

#### Colonic microbiota diversity

**Figure 4:**
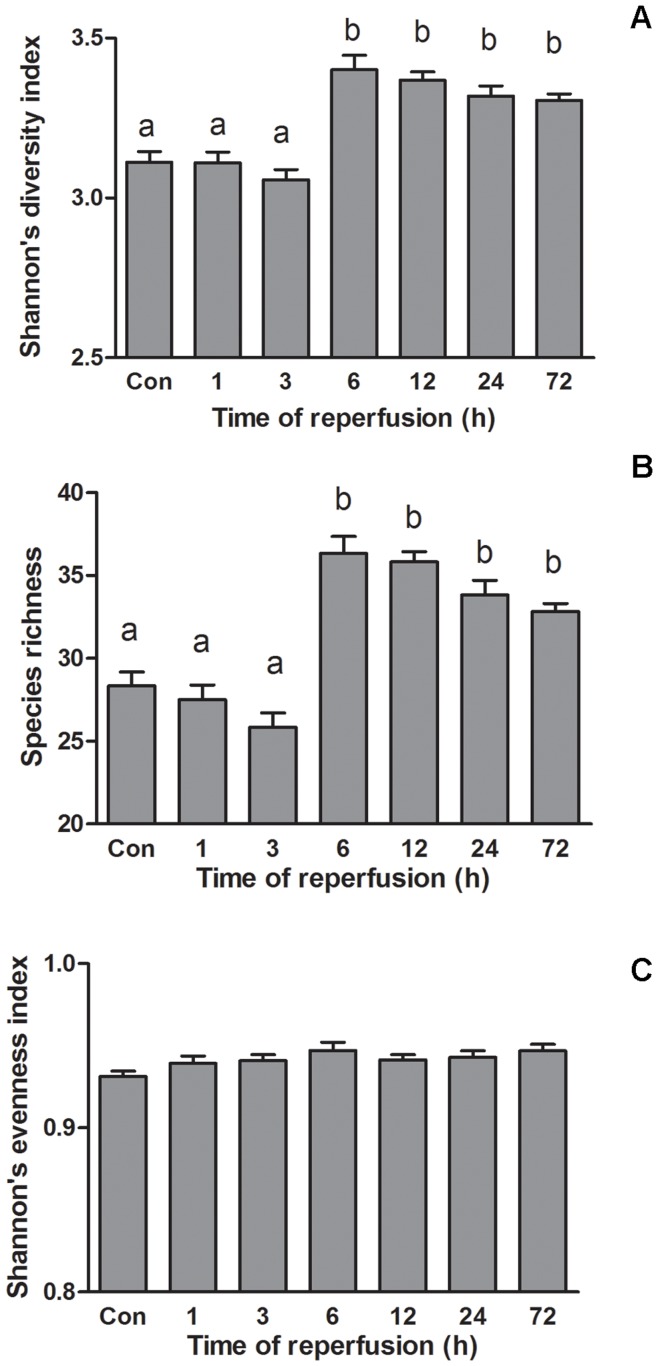
Colonic microbiota dynamic diversity comparison. (A) Shannon’s diversity index. (B) Species richness. (C) Shannon’s evenness index. Columns within class (labeled a or b) not sharing letters are different (*p*<.05).

The species richness was lower in the control, 1 hour and 3 hours reperfusion groups versus the following four time points groups. Means of species richness are 28.33, 27.50 and 25.83, versus 36.33, 35.83, 33.83 and 32.83. A similar result was obtained using the Shannon’s diversity index (Figure 4A and B). There was no significant difference in the colonal microbial evenness for all groups of rats in the experiments (Figure 4C).

#### Bacterial species in relation to intestinal ischemia-reperfusion

**Figure 5:**
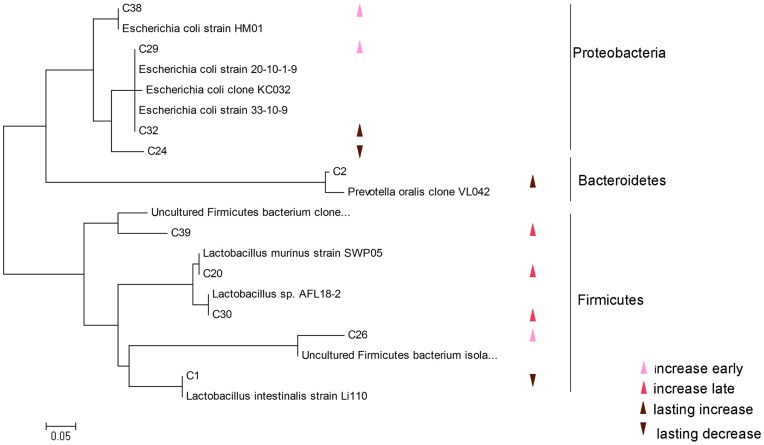
Phylogenetic distribution of meaningful bacterial 16S rDNA sequences and related clones from GenBank. The bar indicates 5% sequence divergence.

To more specifically define changes in the microbiota induced by gut I/R, we identified bacterial species that mainly contributed to the PCA clustering by species scores on axes of PCA analysis results [25]. For example, bands that have largest projections on the positive axis 1 are correlated with the bacterial populations increased in 1 and 3 hours of reperfusion. Therefore, every 3 bands that had the largest absolute values of species scores on each axis were excised from the DGGE gel and sequenced to identify which species they are. The bands were assigned to a bacterial species based on highest (≥90%) sequence similarity match to GenBank sequences obtained by BLAST analysis (Table 1). Band C12 was too weak to be amplified, thus we excised band C20, whose species scores ranked fourth. Band C25 was excluded because of low alignment score, while band C5 was also excluded for no significant similar sequence was found. In general, the microbial shifts occurred in four major patterns (a) bacterial groups that increased early at reperfusion hour 1 and 3 (positive axis 1), two strains of *Escherichia coli* (band C38 and C29) and an Uncultured Firmicutes bacterium (band C26); (b) bacterial groups that increased later, especially characterized at hour 6 (negative axis 1), 2 species of *Lactobacillus* (band C30 and C20) and an Uncultured Firmicutes bacterium (band C39); (c) bacterial groups that increased within a longer time, from reperfusion hour 1 to hour 6 (positive axis 2), a *Prevotella oralis* (band C2) and an *Escherichia coli* (band C32) and (d) bacterial groups that decreased together with groups which increased in pattern (c) (negative axis 2), a *Lactobacillus sp.* (band C1) and an *Escherichia coli* (band C24). These species were subsequently confirmed by clustering analysis with sequences and their closest phylogenetic neighbours (Figure 5).

**Table 1:** Identification of bacteria that characterize colonic microbiota dysbiosis.

Band	Closest BLAST match	Identity (%)	Clone/Strain	Accession no.
C38	*Escherichia coli*	98	HM01	JN811622.1
C26	Uncultured Firmicutes bacterium	93	isolate DGGE gel band UC03-5	JF833128.1
C29	*Escherichia coli*	97	20-10-1-9	JF919881.1
C30	*Lactobacillus sp.*	99	AFL18-2	AB559567.1
C39	Uncultured Firmicutes bacterium	92	CF2-190	GU958647.1
C20	*Lactobacillus murinus*	97	SWP05	HQ668465.1
C2	*Prevotella oralis*	97	VL042	GU424775.1
C32	*Escherichia coli*	99	33-10-9	JF919882.1
C1	*Lactobacillus intestinalis*	100	Li110	JF923643.1
C24	*Escherichia coli*	93	KC032	GU415815.1

#### Quantitative validation of characteristic bacterial species with real-time PCR

**Figure 6:**
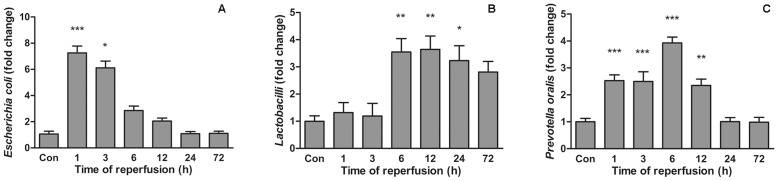
Quantitative analysis of characteristic bacteria species. The relative quantity of specific groups of bacteria was determined by real-time PCR of 16S rRNA gene of (A) *Escherichia coli*, (B) *Lactobacilli* and (C) *Prevotella oralis*, and normalized to total bacteria 16S rRNA gene amount using the 2^–ΔCT^ method. Quantification values are represented by fold changes relative to control rats. ***p<.001, **p<.01 *p<.05.

Quantitative real-time PCR was performed to verify the changes found by DGGE. There was a trend that more *Escherichia coli* were present in 1 and 3 hours of reperfusion (*p*<.001 and *p*<.05 respectively) (Figure 6A). *Lactobacillus* 16S rRNA gene content was not significantly higher until 6 hours of reperfusion (Figure 6B), whereas number of *Prevotella oralis* elevated during 1 hour to 12 hours of reperfusion (Figure 6C). The differences in the quantitative data of the three bacteria groups in the intestinal I/R time course reflect the disproportions identified in the DGGE analysis.

### Colonic Epithelial Barrier Impairment and Recovery Precedes Gut Flora Dysbiosis

#### Damage and repair of colonic mucosa after intestinal ischemia-reperfusion

**Figure 7:**
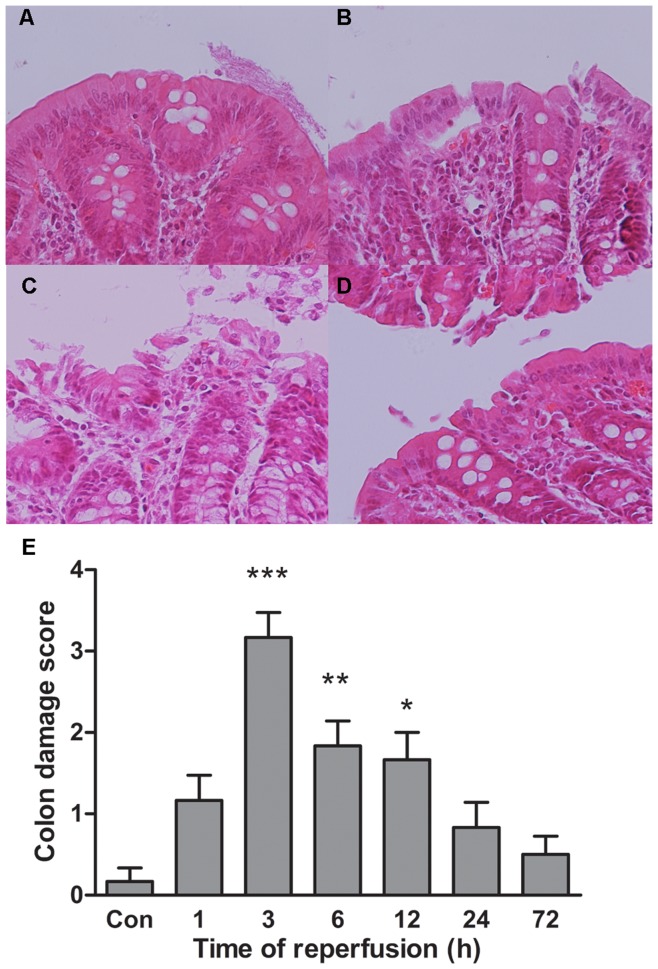
Dynamic changes of colonic epithelial histopathology. (A–D), representative graphs of control, 1-hr, 3-hr and 6-hr reperfusion groups, respectively. (E), comparison of colon damage scores of control group with the grades of different reperfusion time groups. ***p<.001, **p<.01 *p<.05.

The microscopic inspection of colonic mucosa showed focal epithelial edema and necrosis at 1 hour after ischemia and reperfusion (Figure 7B). Three hours of reperfusion group showed the highest injury scores (3.17±0.31) compared with normal group (0.17±0.17, *p*<.001) (Figure 7E). Severe and widespread necroses with hemorrhage were detected at that time (Figure 7C). Whereas at 6 hours of reperfusion, morphological alteration of colonal mucosa tended to recover (Figure 7D). Histological injury scores of ischemia and 24 hours of reperfusion colon were not significantly different from those non-ischemia colon.

#### Serum D-lactate levels

**Figure 8:**
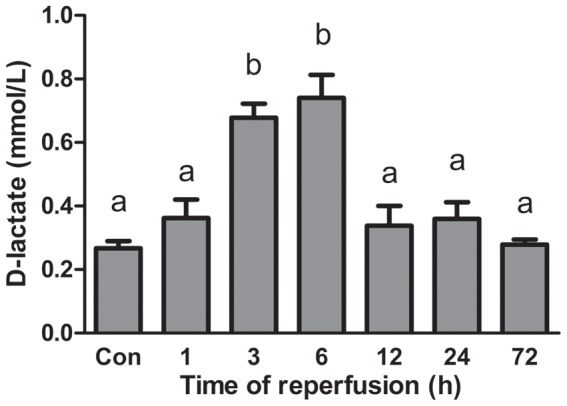
Serum D-lactate levels in intestinal ischemia-reperfusion rats. Columns within class (labeled a or b) not sharing letters are different (*p*<.05) in D-lactate concentrations.

Three to six hours after intestinal ischemia and reperfusion, serum D-lactate levels (0.68±0.04 and 0.74±0.07) were significantly elevated compared with normal and other groups (*p*<.001). After 12 hours of reperfusion, the serum D-lactate concentration (0.34±0.02) dropped back to nearly the normal level (0.27±0.02). (Figure 8).

#### Comparison between timing of gut flora and epithelial barrier changes

Examination of proximal colon and its contents that suffered ischemia-reperfusion demonstrated that earlier damage and repair of the epithelium compared with later dysbiosis and tendency of recovery of colonic microbiota. Colon damage scores and serum D-lactate concentration elevated as early as 1 hour after reperfusion and reach the highest level at hour 3 (Figure 7E and Figure 8), while colonic community’s pattern showed a slight change at those time (Figure 2 and 3). Community’s pattern presented the most prominent change at 6 hours of reperfusion. Epithelial barrier started repairing after 3 hours and gained full recovery at 24 hours of reperfusion. However, the colonic luminal composition did not cluster with normal microbiome during the same time period and neither at 72 hours of reperfusion.

## Discussion

Several reports have demonstrated that intestinal microbiome dysbiosis exits in critical illnesses. However, precisely how and when the composition of gut flora alters after intestinal ischemia-reperfusion remains unclear. In this study, we observed kinetics of colonic flora alteration during rats intestinal I/R, identified distinctive bacteria species in the pathogenesis and showed that colonic epithelial barrier impairment and recovery precedes gut flora dysbiosis.

Our study showed for the first time that intestinal I/R does affect the bacterial construction of colon of rats. Clustering analysis revealed distinct clusters (Figure 2) that we designated as 2 microbiome patterns and 2 sub types within each. This suggests that colonic flora started to change as early as 1 hour reperfusion and reached the most distinct microbial construction at 6 hours reperfusion. The result was then confirmed by PCA, which in addition demonstrated that colonic community pattern began shifted back towards normal pattern after 6 hours reperfusion. To the best of our knowledge, this is also the first time that 16S rRNA based bacterial community profiling has been used to monitor colonic flora change in a model of intestinal ischemia and reperfusion.

We next sought to determine whether the changes of profiling were due to several reduced and/or increased bacteria. Apart from two kinds of uncultured Firmicutes, *Escherichia coli* (band C38 and C29) growth responded to ischemia and reperfusion injury quickly, while *Lactobacilli* (band C20 and C30) tended to proliferate a bit later. Served as a potential pathological bacterium, *Escherichia coli* could increase colonic wall permeability [26] and has pro-inflammation potential on the intestinal tissue [27]. In addition, *Escherichia coli* accumulation could be seen in rodent experimental colitis [27], [28] and resulted in more bacteria translocation [29]. Moreover, our previous studies demonstrated that enteropathogenic *Escherichia coli* invades intestinal epithelial barrier by redistribution of tight junction proteins both in vivo and in vitro [30], [31]. In a similar in vitro model, intestinal epithelial cells incubated with *Escherichia coli* and subjected to hypoxia and reoxygenation would underwent increased deaths [32]. These investigations and the present study reassure that *Escherichia coli* overgrowth plays an important role in gut inflammation and bacteria translocation.

*Lactobacilli* are normal components of the intestinal flora. In our study, we found that *Lactobacilli* increased later in the injury of intestinal I/R. However, in a murine colitis model, numbers of *Lactobacilli* remained unaffected [27], while IL-10 gene deficient mice have deceased levels of colonic *Lactobacilli* [33]. The inconsistency may be explained by model differences. Our model is such a more acute ischemia-reperfusion model that the colonic mucosa started recovering at 6-hr reperfusion. This time point is consistent with the moment *Lactobacilli* accumulated prominently in our study. Likewise, some studies proved that certain strains of *Lactobacillus* may improve the colonic barrier [26], [34], normalizing *Lactobacilli* levels even reduced colonic mucosal adherent, bacterial translocation and colitis injury [33].

Considering the second principal component, *Prevotella oralis* (band C2) proliferation characterizes the community change throughout the first 6 hours. Although *Prevotella oralis* was mostly isolated from the oral cavity [35], this species could be isolated from infected mice intestinal contents [36] and feces of a patient who was prolonged high-dose ciprofloxacin treated [37]. *Prevotella oralis* is a gram-negative, rod-shaped anaerobic bacterium and has been reported translocating to systemic circulation [38], the heart [39] and the lungs [40]. A possible role for *Prevotella oralis* in the pathogenesis of intestinal I/R may be an overgrowth in the injured colon, followed by translocation through the impaired gut wall and causing infection in remote organs. Other bacteria that contribute to the second principal component were two *Escherichia coli* and a *Lactobacillus*. The shifting tendencies of these bacteria are not equal to each other and the ones previous described. The theory that could explain the phenomenon is that bacteria species also compete with their kin in a bacterial ecological community [41].

The acute stage of colonic ischemia and reperfusion was characterized by a shift towards more members of bacteria and much more complexity, whereas a loss of bacterial microflora diversity was seen in IBD colonic mucosa [42]. This phenomenon may be attributed to differences of diseases and flora locations. In addition, in our rat’s model, we can rule out the possibility that antibiotic treatments and nutrition starvation may affect the intestinal microbiota, which is common in critical ill patients underwent intestinal ischemia- reperfusion.

Colonic epithelia showed healing at 6 hours after reperfusion while the gut flora reached the highest diversity and the most different pattern. It indicates that the histological injury precedes gut flora dysbiosis and gut I/R had a longer effect on the luminal microbiota. Observations of the epithelial barrier and gut flora at the same time were novel of this injury model and consistent with the theory that gut is the motor of critical illness [43]. Previous study has shown that host inflammation disrupted intestinal microbiota and supported the growth of Enterobacteriaceae family [28]. The colonic epithelial cells got inflammation after ischemia and reperfusion. It has been proved that inflammation harms colon epithelium’s ability of expressing antimicrobial peptides [44]. Other mechanisms such as pattern recognition receptors, mucin production and IgA secretion might also participate in interactions between the epithelium and gut flora [43].

Although there are several previous studies focused on gut flora changes in critical illnesses [45], molecular analyses based on 16S rRNA gene are few. DGGE is able to identify bacterial populations that constitute 1% or more of the total bacterial community [46]. Therefore, our results demonstrated a new opportunistic pathogen species *Prevotella oralis* that proliferated in gut ischemia and reperfusion as well as *Escherichia coli*. Culture based or quantitative PCR methods only examine species that are designed to detect, which do change but usually do not alter predominantly and contribute significantly in the pathogenesis of the disease.

Together, our study showed that gut ischemia-reperfusion affected the colonic microbiota pattern dynamically and promoted overgrowth of *Escherichia coli* and *Prevotella oralis*. Proliferation of *Lactobacilli* in the later reperfusion phase was concordant with the colonic epithelium recovery time course. In addition, epithelial barrier impairment and repair preceded gut flora dysbiosis and restoration in this injury model.

## Materials and Methods

### Animals, Surgical Procedures and Sample Collection

Male Sprague-Dawley rats (200–250 g) were kept in the Laboratory Animals Center of Jinling Hospital with standard chow and water available ad libitum except during experimental procedures. Every two rats shared one cage. The lights were maintained on a 12-h: 12-h light-dark schedule. All procedures were carried out in accordance with the “Guide for the Care and Use of Laboratory Animals” published by the National Institutes of Health (NIH publication 86–23 revised 1985). And the protocols were approved by Animal Care and Use Committee of Jinling Hospital. Rats were anesthetized with ketamine (100 mg/kg, intraperitoneal). To perform superior mesenteric artery ischemia-reperfusion, a midline laparotomy was made, and then a clamp was placed on the superior mesenteric artery for 30 minutes, after which the tissue is reperfused. Forty-two rats were divided randomly into 7 groups with 6 animals per group. A group of rats were sacrificed just after anesthesia as the control group, while other six groups were euthanized at 1, 3, 6, 12, 24, 72 hours of reperfusion respectively. Blood samples and tissues were harvested for further analysis. Rats were then killed by over-dosed anesthesia. Colonic samples and its contents at 1 cm distal to the cecal-colonic junction, were immersed in liquid nitrogen, and stored at −80°C for further analysis.

### Microbial Analysis of the Proximal Colonal Content

#### DGGE profiling

Extraction of DNA from proximal colonal contents was carried out using the QIAamp DNA Stool Mini Kit (QIAGEN, Hilden, Germany), according to the manufacturer’s instructions, and stored at −20°C until use. To investigate bacterial community composition in extracted DNA, PCR amplifications of V3 variable region of the 16S rRNA gene were carried out using the forward primer (GC357f 5′-CGCCCGGGGCGCGCCCCGGGCGGGGCGGGGGCACGGGGGGATTACCGCGGCTGCTGG-3′) and reverse primer (518r 5′-CCTACGGGAGGCAGCAG-3′). The 50 µl PCR reaction mixture were: 1 µl of extracted bacterial DNA, 5 µl of 10×PCR buffer, 1 µl of dNTP mixture (2.5 mM each), 1 µl of each primer (10 pM), 0.5 µl of *Taq*-Polymerase (5 U/µl), 40.5 µl sterile water. A touchdown PCR was run in an Applied Biosysterm 2720 Thermal Cycler. Initial denaturation was at 94°C for 5 min, amplification was carried out using 20 cycles including denaturation at 94°C for 30 sec, annealing at 65°C for 30 sec decreased by 0.5°C for each cycle, and extension at 68°C for 30 sec. This was followed by additional 10 cycles of denaturation at 94°C for 30 sec, annealing at 55°C for 30 sec, extension at 68°C for 30 sec, and a final extension at 68°C for 7 min.

DGGE was carried out as previously described [15] using the DCode Universal Mutation Detection System (Bio-Rad Laboratories, Hercules, CA, USA). Gels that contained 8% polyacrylamide (ratio of acrylamide to bisacrylamide was 37.5∶1) in 1×TAE buffer (40mM Tris–acetate, 1mM EDTA; pH 7.4) in a gradient of 35%–50% denaturant were used to separate PCR fragments where 100% denaturant was defined as 7M urea and 40% formamide. The electrophoresis was conducted first at 200V for 10 minutes and then at 120V for 7.5 hours in 1×TAE buffer and at a constant temperature of 60°C with the DCode system. All gels were stained with SYBR Green Ι (Invitrogen) for 30 min, and scanned under U.V illumination. Images were captured by ChemiDoc XRS Camera (BIO-RAD).

#### Digital processing of DGGE profiles

On each DGGE gel, two common samples were loaded on both outer sides for digital gel normalization and to allow comparison among gels. Gel images were aligned using Adobe Photoshop CS4, and digitized by Quantity One software (version 4.6). The latter software was used to detect Individual bands in each sample lane, using a match tolerance of 0.5%, followed by manual correction if necessary. Bands occupying the same position across different lanes were matched and identified as the same band type. The relative quantity of a given band was expressed as a proportion (%) of the sum of all defined bands in the same lane. The quantitative information derived from relative band quantities per band type per sample was exported as a data matrix.

#### Comparative analyses of the DGGE profiles

Dice Coefficient was used to calculate similarity between two lanes. Dendrogram was constructed by the unweighted pair group method with arithmetic mean (UPGMA). Principal component analysis (PCA) was performed using Canoco (version 4.5) based on the data matrix mentioned in **Digital processing of DGGE profiles**. Richness was calculated as the total number of each sample lane’s bands. Diversity was calculated using Shannon’s diversity and evenness index with quantitative information. [47].

#### Sequencing of DGGE bands

DGGE bands that differed significantly in different reperfusion time points according to the multivariate analysis were excised with a sterile scalpel and placed into a single Eppendorf tube. The DNA was eluted form the gel slice into 20 µl of sterile water overnight at 4°C. The resulting DNA solution was then amplified again using the V3 16S rRNA forward primer (without GC clamp) (357f 5′- ATTACCGCGGCTGCTGG -3′) and the reverse primer (518r 5′-CCTACGGGAGGCAGCAG-3′). The 50 µl PCR reaction mixture were: 4 µl of template DNA, 5 µl of 10×PCR buffer, 1 µl of dNTP mixture (2.5 mM each), 1 µl of each primer (10 pM), 0.5 µl of Taq-Polymerase (5 U/µl), 37.5 µl sterile water. A touchdown PCR was run in an Applied Biosysterm 2720 Thermal Cycler. Initial denaturation was at 94°C for 5 min, amplification was carried out using 20 cycles including denaturation at 94°C for 30 sec, annealing at 65°C for 30 sec decreased by 0.5°C for each cycle, and extension at 68°C for 30 sec. This was followed by additional 15 cycles of denaturation at 94°C for 30 sec, annealing at 55°C for 30 sec, extension at 68°C for 30 sec, and a final extension at 68°C for 7 min.

Purified PCR products were sequenced using the Sanger’s method on an ABI 3730 automated sequencing system. The retrieved sequences were compared with NCBI GenBank databases using the BLAST tool. Based on the BLAST results, reference sequences of phylogenetic neighbor species (up to 90% similarity) were included for constructing phylogenetic tree using the MEGA 4.0 program in the method of neighbor-joining based on evolutionary distances.

#### Real-time PCR

Bacterial species that differed significantly in the ischemia-reperfusion injury in the DGGE comparative analyses were quantified by quantitative PCR using the 7300 Real-Time PCR System (Applied Biosystems, USA) and the primer pairs specific for the bacteria are shown in Table 2.

**Table 2:** 16S rRNA gene primers for real-time PCR.

Target group	Forward primer (5′- 3′)	Reverse primer (5′- 3′)	Product size (bp)	Reference
Bacteria	CGGTGAATACGTTCCCGG	GGWTACCTTGTTACGACTT;	142	[50]
*Escherichia coli*	CATGCCGCGTGTATGAAGAA	CGGGTAACGTCAATGAGCAAA	96	[51]
*Lactobacilli*	TGGATGCCTTGGCACTAGGA	AAATCTCCGGATCAAAGCTTACTTAT	92	[52]
*Prevotella oralis*	CACRGTAAACGATGGATGCC	GGTCGGGTTGCAGACC	532	[53]

The real-time PCR was carried out in a 20 µl total reaction mixture using 1 µl of DNA, 0.4 µl of each primer, 10 µl 2 × SYBR Green Realtime PCR Master Mix (TOYOBO, Japan) and 8.2 µl sterile water. The amplification program consisted of one cycle of 95 for 5 min, followed by 40 cycles of 95°C for 15 sec, and 55°C (except for *Prevotella Oralis* 60°C) for 60 sec. Species specific bacteria 16S rRNA gene amount was normalized to total bacteria 16S rRNA gene amount using the 2^–ΔCT^ method. Quantification values are represented by fold changes relative to control rats.

### Measurement of Intestinal Permeability

#### D-lactate concentration

Serum levels of D-lactate was assayed using a enzymatic-spectrophotometric method described previously [48] with the kit (GENMED SCIENTIFICS INC. U.S.A.) according to the manufacturer’s instructions.

#### Morphologic analysis of gut injury

At sacrifice, proximal colon was fixed in 10% buffered formalin, embedded, sectioned, stained with hematoxylin and eosin and examined by light microscopy. For each animal, the criteria used to score the degree of colonal injury were based on an established grading system [49]. The numerical scores were the following: 0 =  normal mucosa; 1 =  partial epithelial edema and necrosis; 2 =  diffuse swelling and necrosis of the epithelia; 3 =  necrosis with submucosal neutrophil infiltration; 4 =  widespread necrosis with massive neutrophil infiltration and hemorrhage. The degree of damage was blindly scored.

### Statistical Analyses

Statistical significance among groups for bacterial richness, evenness, diversity, injury score and d-lactate concentration were determined by one-way ANOVA analysis followed by post hoc Bonferroni’s multiple comparison test. For non-parametric data, Kruskal-Wallis followed by Dunn’s multiple comparison test was used. *P* values less than .05 were considered statistically significant. All data were presented as mean ± standard error of the mean. The analyses were performed using GraphPad Prism version 5.
